# Knowledge assessment regarding secondary prevention of coronary heart disease- a multi centre survey

**DOI:** 10.1186/1472-6920-14-113

**Published:** 2014-06-06

**Authors:** Anne Thushara Matthias, Niroshan C Lokunarangoda, Ruvan Ekanayaka

**Affiliations:** 1 University Medical Unit, Colombo South Teaching Hospital, Colombo, Sri Lanka; 2 Institute of Cardiology, National Hospital of Sri Lanka, Colombo, Sri Lanka

**Keywords:** Cardiovascular disease prevention, Coronary heart disease, Secondary prevention, Sri Lanka, Continuous medical education

## Abstract

**Background:**

Cardiovascular Disease (CVD) is a major cause of mortality worldwide. Control and reduction of cardiovascular risk factors such as elevated blood pressure, high cholesterol levels, excess of body weight, smoking and lack of exercise can contribute to a reduction of CVD mortality.

**Methods:**

A standardized questionnaire was administered to all medical officers willing to participate in the study, who were working in the Cardiology Units all over Sri Lanka to assess the source of continuous medical education, attitudes on secondary prevention, barriers to secondary prevention and knowledge assessment of secondary prevention of cardiovascular diseases. Chi square was used to compare groups and p < 0.05 was considered significant.

**Results:**

132 participants with equal numbers of males and female doctors participated. While 56 doctors have had no training in cardiology, 75 doctors have had some training in a cardiology unit. The barriers for secondary prevention were, poor knowledge/understanding of patients 3.82 (1.06), too many drugs 3.74 (0.98), presence of co-morbid conditions 3.68(0.97), cost of medications 3.69 (0.97) and poor adherence to prevention strategies by patients 3.44 (1.15). Routine clinic visits 85 (65%) and public awareness day seminars 30 (22.2%) were the most effective methods of secondary prevention. Guidelines were the most popular method of continuous medical education. Those who have had some training in cardiology did not differ in their knowledge from those who have never had training in cardiology. Knowledge about prevention with regard to diet was inadequate and exercise and lipids were adequate but not good. Rates of knowledge on smoking cessation were much higher than for other CVD risk factors.

**Conclusion:**

There needs to be more adherences to clinical guidelines and attention paid to CVD prevention, in particular, the importance of dietary modifications, adequate exercise, and lipid control.

## Background

Cardiovascular Disease (CVD) is a major cause of mortality worldwide. Studies have proven that control and reduction of cardiovascular risk factors such as elevated blood pressure, high cholesterol levels, excess of body weight, smoking and lack of exercise can contribute to a reduction of CVD mortality of around 40-60% [1].

To optimally manage the risk factors for CVD, the risk factors must be identified. This will enable risk stratification of patients with CVD. There are different risk estimation scores: SCORE [2], Framingham [3] and ASSIGN–SCORE [4]. Risk factor estimations are important as atherosclerosis is a multifactorial disease. These risk estimation scores are built on the ideal target levels for each risk factor. The targets are values at which each risk factor control is started. A group of modestly deranged risk factors can result in a higher total risk than a single deranged risk factor as multiple risk factors give multipliable and additive risk [5]. A clinically useful CVD risk-estimation system should be methodologically robust, easy to use and should have a beneficial outcome [5]. Though risk factor estimation systems should theoretically improve outcomes, not many studies have proven the above [5]. Therefore as of now, until the actual benefits of risk-factor estimation systems can be proven, everyday clinical practice should involve the assessment of risk factors and control of them. In order to control risk factors one needs to be aware of the target level for each factor. Though at present there are no studies done to prove that knowing the targets for each risk factor is beneficial, knowing the targets for each risk factor would enable physicians to participate to secondary prevention in an active way. The concept of controlling each risk factor at a particular threshold is a controversial concept as CVD risk is a continuum [6]. Yet until further trials are available comparing the effects of controlling individual risk factors versus total risk factor control of a patient, it is wise to control a risk factor once identified.

The targets for risk factors control change from time to time. They are the result of extensive of clinical research. In an attempt to standardize and improve care, international health agencies have published guidelines for secondary prevention of cardiovascular risk factors. The European Society of Cardiology and American Heart Association [7], National High Blood Pressure Education Program committee (JNC 7) [8], and National Cholesterol Education Panel (ATP III) [9] are some of them.

Studies assessing the knowledge and efforts of doctors in secondary prevention have found that doctors do not stress enough on the role of secondary prevention to their patients [10]. Lack of awareness about prevention guidelines [11] and lack of motivation [12] could be possible reasons. The lack of adherence to guidelines leads to erroneous or suboptimal management of risk factors.

The objectives of this study were to assess the knowledge on secondary prevention among medical officers, to find out the methods of Continuous medical education (CME) among medical officers and to find out their perceived role in risk factor prevention.

CME is one powerful method of bridging the gap between evidence based medicine and clinical practice. Ongoing CME is an important area for the maintenance of good quality clinical care [13]. In most countries in South Asia, though there are various continuing medical education programs, there is no revalidation process and no established system of awarding credits for educational activities [14]. Sri Lanka is a developing country and there is no established compulsory continuous medical education program or revalidation process in Sri Lanka at present. There is a National Continuous Professional Development Program, which is voluntary [14,15]. There is a dearth of data from Sri Lanka on the impact of continuing medical education programs in general. No studies have been done on knowledge assessment on secondary prevention of cardiovascular disease in Sri Lanka. This study hopes to fulfill the above objectives with the aim of better secondary prevention of cardiovascular risk factors in the country.

## Methods

Ethical clearance was obtained from the Ethical Review Committee of the National Hospital of Sri Lanka (NHSL). A standardized questionnaire (Additional file 1: Questionnaire) was administered to all medical officers who were working in the Cardiology Units all over Sri Lanka and the medical officers working in the medical wards of the National Hospital of Sri Lanka. Informed consent was obtained. All participants who were willing to take part were administered the questionnaire.

### Development of questionnaire

The questionnaire consisted of three parts.

Part 1 - Demographic details of the participants

Part 2 - Source of CME

Part 3 - Attitudes on secondary prevention. Barriers to secondary prevention were given and the participants were asked to rate on a 1–5 likert scale. 1 been not a significant barrier and 5 been very significant barrier.

Part4 - Knowledge assessment of secondary prevention. This part was further subdivided in to prevention of smoking, blood pressure control, lipids, dietary management, alcohol use, physical activity, weight, management of diabetes, drugs therapy. This included questions regarding the targets for optimal control for each of the above and important questions on prevention. They were open ended questions.

The questionnaire was developed by the authors who included a Senior Consultant cardiologist. The targets expected were based on the present guidelines applicable at the time of study namely: AHA/ACCF Secondary Prevention and Risk Reduction Therapy for Patients With Coronary and Other Atherosclerotic Vascular Disease: 2011 Update [7], National High Blood Pressure Education Program committee (JNC 7) [8], and National Cholesterol Education Panel (ATP III) [9] and ACCORD trial.

Statistical analysis was done using SPSS V.17.0 (SPSS inc. Chicago, IL). Chi square and t test was used for data analysis. The level of significance was <0.05.

## Results

### Participant characteristics

A total of 132 participants with equal numbers of male and female doctors responded to the questionnaire. The majority of the participants were in the age group of 30–40 years. The mean age was 33. The number of doctors in a post graduate training program (Registrars and Senior Registrars) was 41. While 56 doctors have had no training in cardiology, 75 doctors have had some training in a cardiology unit. The demographic details are given in Table 1.

**Table 1:** Demographic data

**Characteristic**	**Number**
**Sex**	
Male	66(50)
Female	66(50)
**Age**	
<30	32(24)
30-40	71(54)
>40	17(13)
**Station of work**	
Anuradhapura	4(3)
Colombo North Teaching Hospital	1(0.7)
Colombo South Teaching Hospital	9(6.8)
Matara	5(3.7)
National Hospital of Sri Lanka (NHSL)	86(65.1)
Peradeniya	1(0.7)
Sri Jayewardenepura General Hospital	4(3)
Jaffna	5(3.7)
Kandy	1(0.7)
Karapitiya	17(12.8)
**Years in service**	
<3 years	51(38.7)
4-6	43(32.5)
7-9	7(5.3)
10-13	29(21.9)
14-16	1(0.7)
**Training/working in cardiology unit**	
No training	56(42.4)
<1 year	47(35.6)
1- 4 years	20(15.15)
>4 years	9(6.8)
**Rank**	
Inter medical officer	26(19.6)
Medical officer	66(0.5)
Registrar	30(22.72)
Senior registrar	11(8.3)

### Awareness about guidelines and barriers to adherence

Among the leading reasons the participants sighted as barriers for secondary prevention were, poor knowledge/understanding of patients 3.82 (1.06), too many drugs 3.74 (0.98), presence of co-morbid conditions 3.68(0.97), cost of medications 3.69 (0.97) and poor adherence to prevention strategies by patients 3.44 (1.15). The barriers to guideline adherence given by participants are listed in Table 2.

**Table 2:** Data on secondary prevention

	**Number(Percentage)**
**Method of secondary prevention**	
Routine clinic visits	85(65%)
Public awareness day seminars	30(22.2%)
Visits to General practioners	15(11.1%)
Consultations with consultant cardiologists	3(2.2%)
**Barriers to secondary prevention**	Mean(Standard deviation)
Adverse effects of drugs	2.51(1.10)
Patient adherence	3.44(1.15)
Presence of co-morbid conditions	3.68(0.97)
Cost of medications	3.69(0.97)
Too many drugs	3.74(0.98)
Poor knowledge/understanding of patients	3.82(1.06)
Not enough time	2.76(1.23)
Lack of knowledge of doctor	2.58(1.15)
Recommendation on prevention are unclear	2.62(1.00)
Receive little or no training in prevention	2.96(1.10)
Not interested in prevention	3.07(1.21)
Value acute care more than preventive care	3.25(1.26)
**Sources of CME**	Number(Percentage)
Research papers	42(31.1)
Guidelines	95(70.4)
CME	51(37.8)
Consultations with specialists	49(36.3)

Routine clinic visits 85 (65%) and public awareness day seminars 30 (22.2%) were the most effective methods of secondary prevention. Guidelines were the most popular method of continuous medical education among the participants (Table 2).

### Lifestyle, smoking recommendations and lipids, diabetes and blood pressure management

The knowledge and adherence to guideline recommendation on lifestyle modification, smoking prevention, lipid, blood pressure and diabetes management are given in Table 3.

**Table 3:** Knowledge on secondary prevention and knowledge among those who has training versus those who did not have training in cardiology

**Target**	**Percentage of doctors identifying correct targets**	**No training in cardiology**	**Training in cardiology**	**P value**
**Tobacco consumption/smoking**				
Ask about tobacco use status at the initial visit	128(94.8)	53	73	0.657
Advise every tobacco user to quit	124(91.9)	51	71	0.069
Assist by counseling and developing a plan for quitting or pharmacotherapy (including nicotine replacement and bupropion)	53(39.3)	16	36	0.068
Arrange follow up, referral to special programs	52(38.5)	27	25	0.068
Questions regarding passive exposure to smoke	70(51.9)	30	39	0.856
**Blood pressure targets**				
What is the systolic blood pressure (SBP) target for patients without Diabetes mellitus (DM) or Chronic kidney disease (CKD)	60(44.47)	23	36	0.593
What is the DBP target for patients without DM or CKD	53(39.3)	22	30	0.716
What is the target for patients with DM or CKD	60(44.4)	21	39	0.365
What is the target for patients with DM or CKD	18(13.3)	23	41	0.366
**Lipid Lowering targets**				
What is the goal of low density lipoproteins (LDL) for secondary prevention?	55(40.7)	23	31	0.090
What is the goal of non high density cholesterol (HDL) cholesterol if triglyceride (TG) > 200 mg/dl?	14(10.4)	3	11	0.090
What is the goal of non high density cholesterol (HDL) if triglyceride (TG) > 200 mg/dl?	18(13.3)	7	11	0.254
What is the dose of Atorvastatin you would prescribe?	0	10	11	0.354
40 mg	0	0	0	0
80 mg				
What is the level of TG at which you would initiate therapy with Niacin/Fibrate before statin?	21(15.6)	9	12	0.270
**Fat intake in diet**				
How much of fat can an average adult take for a day in grams?	4(3)	0	0	0.562
What is the % of polyunsaturated fatty acids (PUFA) out of total fat intake?	14(10.4)	4	9	0.393
What is the % of monounsaturated fatty acids (MUFA)	8(5.9)	0	0	0.007
What is the % of Saturated fatty acids?	12(8.9)	2	6	0.465
What is the amount of recommended daily salt intake in grams/teaspoons?	37(27.4)	14	22	0.561
**Alcohol intake**				
What is the amount of alcohol which is safe to consume for men?	49(36.3)	4	15	0.000
What is the amount of alcohol which is safe to consume for women?	51(37.8)	2	16	0.000
**Physical exercise**				
What is frequency of exercise per week?	40(29.6)	27	27	0.214
What is the duration per session?	79(58.5)	34	56	0.089
**Waist circumference and weight**				
What is the optimal waist circumference for men?	33(24.5)	18	15	0.578
What is the optimal waist circumference for women?	28(20.7)	15	15	0.157
What is the goal of HBA1c?				
6.9	1(0.7)	1	0	0.520
7	62(45.9)	32	30	0.217
**Drug therapy**				
Is ACEI indicated in all patients with EF < 40% with no contraindication?	89(65.9)	33	56	0.652
Would you prescribe beta blockers to all patients after ACS if there are no contraindications?	113(83.7)	44	68	0.483
Aspirin duration after an episode of acute coronary syndrome	83(61.5%)	40	57	0.654
Aspirin dose after an episode of acute coronary syndrome	99(73.4%)	28	53	0.088
Clopidogrel dose after an episode of acute coronary syndrome	100(74%)	38	61	0.073
Clopidogrel duration after an episode of acute coronary syndrome	29(21.5%)	5	5	0.074

A modest number of participants 40(29.6%) advised patients to engage in physical activity more than 5 times a week as recommended by the guidelines. The minimum duration of 30 minutes was also a well known factor among this group of doctors (58.5%).

A significant proportion of participants identified blood pressure targets set up by the AHA for general population, but the targets for patients with Diabetes and Kidney disease were not identified as frequently as the above.

About 40.7% participants identified the LDL target of 100 mg/dl as ideal but only 18% were able to give the correct dose of Atorvastatin for treatment. There was moderate understanding about triglyceride level management as only 21% were able to give the target at which treatment should be started before a statin.

Majority of doctors did not specify the exact targets for fat intake. The overall response rates for fat intake been all less than 10.4%. The recommended alcohol intake for men (36.3%) and women (37.8%) both were identified by a significant number of participants.

The waist circumference cut off specific for Asians were identified by a 24.5% and 20.7% of doctors. The goal of HBAIC of 7 was correctly identified by 62 (5.9%) of the participants.

The use of Aspirin and Clopidogrel was well known among the participants and there was a response over 80% about the correct dose and duration of Aspirin. The knowledge about the duration of Clopidogrel was only 29%.

When comparing the two groups of participants, those who have had some training in cardiology versus those who have never had training in cardiology, there was no difference in the level of knowledge.

## Discussion

There are issues with regard to the generizability of studies done on knowledge assessment. This preliminary study on the knowledge on secondary prevention of cardiovascular diseases among medical officers is the first of its kind done in Sri Lanka and it reveals several important findings. The findings are relevant in three areas: the level of knowledge on secondary prevention among medical officers, strategies and obstacles to secondary prevention and methods of CME used by doctors.

### Knowledge on secondary prevention

Counseling may be an effective tool in reducing behavioral risk factors for CVD. Yet, doctors do not counsel their patients aggressively about lifestyle changes to prevent CVD. In our study the knowledge about prevention with regard to diet was inadequate and exercise and lipids were adequate but not good. Rates of knowledge on smoking cessation was much higher than for other CVD risk factors, a finding which has been reported by others [16]. Only a handful of physicians knew about the exact dietary recommendations for fat and salt and the frequency of exercise needs. Over the past few decades there has been a consistent need for the change of dietary habits to prevent cardiovascular deaths. This has been addressed in many leading papers on the subject [17]. Yet the knowledge on the dietary recommendation still remains poor. This calls for a more vigorous shift in the attitudes and knowledge of doctors on dietary recommendations. Over 90% asked about tobacco use at the initial visit and over 50% advised on quitting smoking. The knowledge on secondary prevention of smoking was excellent.

Our study also indicated there is more education needed in blood sugar control targets and blood pressure targets as evidence by a similar study done in USA [18] Blood pressure control plays a vital role in the prevention of CVD. In a recent meta-analysis it was found that blood pressure reduction of 10 mmHg systolic or 5 mmHg diastolic reduced the rate of coronary heart disease by 22% [19].

Lower the serum LDL-cholesterol level, the better it is for cardiovascular disease prevention [20]. Yet not many doctors identified the importance of proper lipid control and they were not certain about the targets for lipid control. The kowledge on lipids was inadequate. This was also revealed by a study done by Freedman et al. In another study done in Germany the knowledge on lipid control was similar to our study [16]. There need to be more educational activities to ensure doctors practice the lipid control guidelines more accurately.

The benefit of Clopidogrel for secondary prevention after an episode of acute coronary syndrome had been shown by many multicentre clinical trials [21]. Yet the duration of Clopidogrel use is not very clear in literature and our respondents were doubtful about the appropriate duration of it. This shows that more emphasis needs to be made on the introduction of evidence based medicine to our setting.

The limited adherence shown to clinical guidelines has been termed clinical inertia. The reasons that could contribute to inertia are doctors overestimating the quality of the care they actually provide, lack of training, and use of “soft excuses” to avoid intervention [22]. In our study we found there was very little clinical inertia when it came to prescribing beta blockers and ACE inhibitors, two drug classes which have morbidity and mortality benefit cardiovascular disease prevention. In our study most doctors (>60%) knew the exact indications for beta blockers and ACE Inhibitors. It is therefore safe to assume they would be prescribing it to all patients who need both the classes of drugs.

This study also showed that awareness and incorporation of CVD prevention did not differ by the training doctors received. The doctors who have not received cardiology training were equally knowledgeable as those who hadn’t received training. Our study explored whether physician training level was predictive of adherence to guidelines. The data form Friedman et al. found that attending physicians and resident physicians did not differ in prevention counseling. The reasons given were that attending physicians did not believe in physician counseling and also were not very knowledgeable in the present guidelines [23].

### Strategies of secondary prevention

There is gap in what is mentioned in clinical guidelines and what is practiced in the sphere of secondary prevention in CVD. The real issue in cardiovascular medicine is implementation of these recommendations into clinical practice [24]. In an attempt to bridge this gap, heath care providers and patients both need to take action [25]. Though there are several available methods of secondary prevention, not many are evidenced based. Until such time evidence becomes available it is wise to take in to account what the physicians think are the most appropriate methods and utilize them to educate patients. When asked from doctors in our study clinic visits were the best approach to educate patients. This is in contrast to the popular belief that a one to one consultation with a consultant or specialist is more useful. Regular clinic visits allowed a proper follow up plan to be implemented. The value of clinics in secondary prevention has been reported elsewhere in the world. A day clinic where patients are educated regarding secondary prevention has found to be very effective [26]. Yet the difference in these clinics done elsewhere in the world, they have less volume of patients, the clinic is primarily focused on secondary prevention and special lectures on atherogenesis and nutrition and an individualized care plan is made at the end of the day. To uplift the status of the secondary prevention in our country will be a difficult task only through clinics because our clinics cater to a massive number of patients, limited time available to devote for each patient and the clinics are mainly run by doctors with no involvement of nursing staff who could be useful in advising patients.

Therapeutic patient education [27] might be one method a country like Sri Lanka should try implementing. Sri Lanka has Cardiac Rehabilitation Programs in several leading hospitals. These have proven to be effective which indicates patients should be and can be used effectively for secondary prevention of CVD [28].

### Barriers to secondary prevention

Over time, studies done all over the world have highlighted several obstacles for optimal cardiovascular disease prevention. The main obstacles identified were lack of time [23], lack of understanding of patients about the disease and adherence to life style modifications [18], the high cost of medication and inadequate time for counseling [29]. The main barriers to secondary preventions as seen by doctors in our study were: poor knowledge of the patients, too many drugs and high cost of medications and the fact that patient values acute care more than preventive care. These barriers were common to Europe as well as Asia as evident by the REACT study [23,30]. Sri Lanka is still a developing country and the majority of patients who utilize the free health care system are not advanced enough to look up and read about their illnesses and learn about them. Poor knowledge of patients was considered a major problem by our respondents. Lack of knowledge is a significant barrier in developing nations [30]. Patients participation been crucial to CVD prevention has been highlighted by studies before [16,18]. In order to overcome the barriers in our setting several important measures could be implemented. We can learn from studies which have proven the efficacy of these methods [30]. To educate patients, patent information leaflets can be utilized. Self care diary maintenance can be promoted. Sri Lanka at present does not have an official referral and counter referral system and any patient can seek treatment from any government or private sector health care facility as and when they wish. This leads to overcrowding of tertiary care centers which leads to less time spent on patients. An organized referral and counter referral system if implemented can bring a drastic improvement in the patient care system as duplication of work will be avoided and patients will be given more care which is continuous.

### Continuous medical education

Practice guidelines were found to be the most common method of CME in our study. This is in contrast to some studies done previously [31]. CME resources are one of the second commonest sources of knowledge in our study population. CME was preferred over clinical practice guidelines in studies done elsewhere [20]. CME programs are always found to be useful to educate and keep the practicing medical officers up-to-date on the new developments in the field. This highlights the need for improvement in the CME programs in the country [32].

### Limitations and future directions

Our study was small. The questionnaire was not subjected to validity tests. The results may not be generalizable to all physicians in the country. Our study also lacked the power to examine whether barriers to treatment varied by age, gender, or race/ethnicity of the physician. However, since our findings indicate a low level of adherence to guidelines, this adds weight to the concern that doctors may be under counseling for CVD prevention. Further larger studies should be carried out to follow up patient’s long term and determine the effect of risk factor control on morbidity and mortality of CVD.

## Conclusion

Overall, this study highlights the need for more CME among doctors to improve secondary prevention of cardiovascular diseases. It also highlights the need for a more robust setup at state hospitals to incorporate nursing staff and other health professionals in running clinics. This might help provide a more qualitative service to patients. Passive dissemination of information is not enough for cardiovascular prevention. Logistically and financially there needs to be a system in place so physicians can exercise proper secondary prevention activities and as highlighted before by many studies more aggressive CME are needed and the mere existence of guidelines will never be enough.

In summary, our study adds to the growing body of literature there needs to be more adherences to clinical guidelines and attention paid to CVD prevention, and, in particular, the importance of diet, exercise, and lipid control. Most importantly, our study did not find any differences in counseling practices between those who have training in cardiology versus those who did not have training finding that highlights the need to create more educational activities to educate both groups.

## Abbreviations

CVD: Cardiovascular disease; CME: Continuous medical education; SBP: Systolic blood pressure; DM: Diabetes mellitus; CKD: Chronic kidney disease; LDL: Low density lipoproteins.

## Competing interests

The authors declare that they have no competing interests.

## Authors’ contributions

All authors were involved in planning, data collection, analysis of data and writing the manuscript. All authors read and approved the final manuscript.

## Pre-publication history

The pre-publication history for this paper can be accessed here:

http://www.biomedcentral.com/1472-6920/14/113/prepub

## Supplementary Material

Additional file 1 Questionnaire. Click here for file
